# Effects of the Fourth Ventricle Compression in the Regulation of the Autonomic Nervous System: A Randomized Control Trial

**DOI:** 10.1155/2015/148285

**Published:** 2015-06-14

**Authors:** Ana Paula Cardoso-de-Mello-e-Mello-Ribeiro, Cleofás Rodríguez-Blanco, Inmaculada Riquelme-Agulló, Alberto Marcos Heredia-Rizo, François Ricard, Ángel Oliva-Pascual-Vaca

**Affiliations:** ^1^Madrid Osteopathic School, Avenida Dr. Heitor Penteado 815, Taquaral, 13075-185 Campinas, SP, Brazil; ^2^Department of Physical Therapy, Faculty of Nursing, Physiotherapy and Podiatry, C/Avicena s/n, University of Sevillla, 41009 Sevilla, Spain; ^3^Department of Nursing and Physiotherapy, University of the Balearic Islands, Carrera de Valldemossa, km 7'5, 07122 Palma, Spain; ^4^University Institute of Health Sciences Research (IUNICS-IdISPa), University of the Balearic Islands, Carrera de Valldemossa, km 7'5, 07122 Palma, Spain; ^5^Madrid Osteopathic School, C/San Félix de Alcalá 4, Alcalá de Henares, 28807 Madrid, Spain

## Abstract

*Introduction*. Dysfunction of the autonomic nervous system is an important factor in the development of chronic pain. Fourth ventricle compression (CV-4) has been shown to influence autonomic activity. Nevertheless, the physiological mechanisms behind these effects remain unclear. *Objectives*. This study is aimed at evaluating the effects of fourth ventricle compression on the autonomic nervous system. *Methods*. Forty healthy adults were randomly assigned to an intervention group, on whom CV-4 was performed, or to a control group, who received a placebo intervention (nontherapeutic touch on the occipital bone). In both groups, plasmatic catecholamine levels, blood pressure, and heart rate were measured before and immediately after the intervention. *Results*. No effects related to the intervention were found. Although a reduction of norepinephrine, systolic blood pressure, and heart rate was found after the intervention, it was not exclusive to the intervention group. In fact, only the control group showed an increment of dopamine levels after intervention. *Conclusion*. Fourth ventricle compression seems not to have any effect in plasmatic catecholamine levels, blood pressure, or heart rate. Further studies are needed to clarify the CV-4 physiologic mechanisms and clinical efficacy in autonomic regulation and pain treatment.

## 1. Introduction

Dysfunction of the autonomic nervous system is considered to be an important factor in the development of chronic pain [1]. Studies using muscle blood flow, heart rate, and blood pressure as outcome measures have suggested an excessive sympathetic activation in patients with different pain conditions such as fibromyalgia [2, 3], musculoskeletal and myofascial pain [1, 4], and chronic pelvic pain [5] and patients who had undergone abdominal surgery [6]. The relationship between sympathovagal balance and psychological distress has also been proven in children with abdominal pain, irritable bowel syndrome [7, 8], and complex regional pain syndrome [9, 10].

Catecholamines are important hormones which regulate ANS activity in both central and peripheral sympathetic nerve endings [11]. Studies with animals have suggested that chronic adrenergic stimulation or impaired epinephrine homeostasis may contribute to the pathophysiological mechanisms of pain syndromes [12, 13]. On the other hand, dopamine has proved to have antinociceptive effects both in animals and in humans [14–16] and dopaminergic neurotransmission has been shown to be affected in chronic pain syndromes [17, 18].

Fourth ventricle compression (CV-4) is a cranial manipulation technique aiming at influencing on brain and cranial nerve function [19]. Some authors have suggested that CV-4 may produce changes in the regulation of the autonomic nervous system (ANS). For instance, Cutler et al. [20] reported muscle sympathetic nerve activity after applying the CV-4 technique. The use of CV-4 technique has also been purported to modify heart rate, blood pressure, blood flow velocity [21, 22], and cerebral tissue oxygenation [23]. Moreover, CV-4 has been observed to produce an increment of electroencephalography alpha absolute power [19], reduce sleep latency [20], and decrease anxiety [21] in healthy subjects. In addition, CV-4 has been shown to be effective in pain relief in tension type headaches [24]. Due to the established interactions between the autonomic and nociceptive systems, the potential effects of CV-4 on the autonomic regulation could represent a novel approach to control the development or maintenance of pain. Nevertheless, the physiological mechanisms undergoing CV-4 autonomic regulation remain unclear. The aim of the present study was to assess the immediate effects of the CV-4 technique compared to sham placebo intervention in plasma catecholamine levels, heart rate, and blood pressure on healthy adults.

## 2. Methods

### 2.1. Participants

Forty healthy adults (19 males and 21 females; age range = 18–33 years) were recruited in the Faculty of Physiotherapy of UNILUS (Santos, SP, Brazil). A member of the research group did a personal interview with every volunteer informing them of the objectives of the study and the procedure. The established exclusion criteria were (1) any local or systemic pathologic condition (recent fracture in the cranial base, osteitis, tumor, encephalopathy, stroke, traumatic brain injury, depression, or asthma crisis), (2) arterial hypotension, low heart rate, or parasympathetic state, (3) pregnant women or women in menstrual state on the evaluation day, (4) tobacco or drug consumption, and (5) having received a manual therapy procedure in the last month. Subjects participating in the study were instructed not to consume coffee, tea, chocolate, vanilla, fruit or fruit derivates, soft drinks, or alcohol for 4 hours before data collection. Subjects who reported having consumed any of these foods were excluded from the study (*n* = 1).

The study was approved in accordance with the principles of the Declaration of Helsinki by the Ethics Committee of Hospital Guilherme Álvaro de Santos, SP, Brazil.

### 2.2. Procedure

Intervention and assessments were performed in a university-based physiotherapy research clinic at Lusíada University (UNILUS, Santos, SP, Brazil). The assessments and intervention were performed between 7 and 9.30 a.m., in a room with a temperature of 21–23°C.

**Figure 1:**
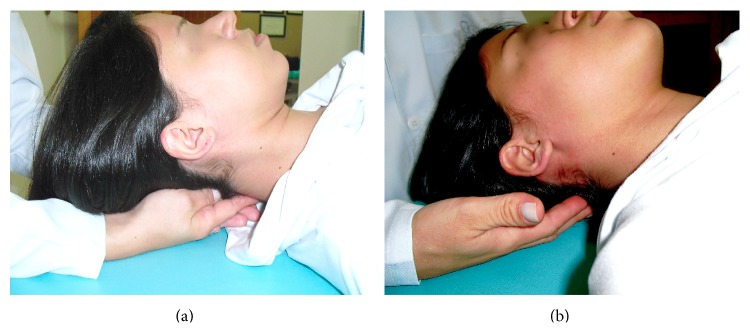
Physiotherapist's hand position during the procedure. (a) CV-4 technique, (b) placebo technique.

Participants were assigned randomly to one of the treatment groups: intervention group and control group. The intervention group received the CV-4 technique for 10 minutes. The CV-4 procedure was administered by an experienced physiotherapist and member of the research group according to the standards established previously in the literature [19]. The participant lay down in a supine position. The physiotherapist, situated behind the participant's head, made a slight approximation of the occipital squama lateral angles towards the posterior occipital convexity, while taking the cranium into extension. The traction was maintained until a motionless state was perceived in the cranial pulse and released when a perception of movement was noted. The procedure was repeated until the 10-minute session time expired (Figure 1(a)).

The control group underwent a bilateral nontherapeutic contact on the occipital bone for 10 minutes. The participant lay down in a supine horizontal position. The physiotherapist, situated behind the participant's head, overlapped their hands touching the occipital bone and provided random slight touch of random duration until the end of the 10-minute session time.  Figure 1(b) displays physiotherapist's hands' position in the control procedure.

### 2.3. Assessments

Both groups (intervention and control group) underwent assessments of blood pressure, heart rate, and blood test to determine plasmatic catecholamine levels before and 5 minutes after the procedure. Blood pressure and heart rate assessments were performed by the same evaluator and the blood test was performed by a second evaluator. Both evaluators entered the room only during the assessment time and were not aware which intervention was done on the participant. Before the intervention, the subject was instructed to sit on a chair in a comfortable position for 10 minutes; after this time, evaluators entered the room and started the assessment protocol. Evaluations were made of blood pressure, heart rate, and blood test, following this order. After the intervention, the participant was instructed to relax in a seated position for 5 minutes before postintervention evaluations were performed.

Plasmatic catecholamines levels were obtained by an analysis of every participant's blood sample. Epinephrine, norepinephrine, and adrenaline were measured according to the methods described by Sealey [25].

Blood pressure was determined by the use of a mercury sphygmomanometer (UNITEC). The assessment was performed 3 times in each arm, with 1-minute interval between them. The average of all measurements was made to obtain systolic and diastolic blood pressure scores.

Heart rate was assessed by stethoscope (Littmann 3M) that was placed on the participant's thorax, over the left heart ventricle. Heart rate was assessed three times with an interval of 1 minute between the measurements. An average of the three measurements was computed to obtain the heart rate score for study purposes.

### 2.4. Statistical Analysis

Analyses of variance (ANOVA) were performed to assess changes in heart rate, blood pressure, and plasmatic catecholamine levels, with GROUP (intervention versus control) as between-subjects factor and TIME (preintervention versus postintervention) as within-subjects factor. Post hoc analyses were performed using post hoc Bonferroni corrected test for multiple comparisons. Analysis was performed with STATA version 7.0. (StataCorp 2001, Stata Statistical Software: Release 7.0, College Station, TX: Stata Corporation, Texas, USA). Significance level was set at *P* < .05.

## 3. Results

Nineteen healthy adults were included in the intervention group (7 females, mean age = 23.58 ± 4.19 years) and twenty healthy adults were in the control group (14 females, mean age = 20.05 ± 1.96 years).  Table 1 displays the values of plasmatic catecholamines, blood pressure, and heart rate for both groups before and after the procedure.

Dopamine plasmatic levels displayed a significant interaction GROUP × TIME (F(1.37) = 7.16, *P* < .05). The control group had lower dopamine levels than the intervention group in the preintervention assessment (post hoc pairwise comparison *P* < .01). Only the control group showed a statistically significant increase in dopamine levels when comparing postintervention scores to preintervention values (post hoc pairwise comparison *P* < .01), whereas no significant changes were seen in the intervention group (post hoc pairwise comparison *P* > .82). Moreover, a main effect TIME (F(1,37) = 6.97, *P* < .05) was found.

Norepinephrine levels witnessed a significant main TIME effect (F(1,37) = 4.57, *P* < .05), showing a decrease of plasmatic norepinephrine levels when comparing the mean score changes from postintervention to preintervention. No significant statistical effects were found for the main effect GROUP (F(1,37) = 2.34, *P* = .14) or the interaction GROUP × TIME (F(1,37) = 1.89, *P* = .177). Nevertheless, pairwise comparisons found that the control group showed lower norepinephrine plasmatic levels in the postintervention assessment, compared to the preintervention values (*P* < .05), whereas no changes were found in the intervention group (*P* = .60). Even though both groups had similar norepinephrine baseline levels (*P* = .65), pairwise comparisons after intervention showed a nonsignificant tendency for the control group to have lower norepinephrine levels than the intervention group (*P* = .052).

Likewise, no significant main effects were found for epinephrine (TIME (F(1,37) = .32, *P* = .36); GROUP (F(1,37) = 2.51, *P* = .12)). Although the interaction GROUP × TIME was not statistically significant (F(1,37) = .87, *P* = .58), post hoc pairwise comparisons showed lower baseline epinephrine levels for the intervention group than for the control group (*P* < .05), whereas no significant differences were found in the postintervention evaluation (*P* = .65). Pairwise comparisons did not show significant changes between the preintervention and postintervention levels for any of the study groups (*P* > .30 in all cases).

Systolic blood pressure had a significant main TIME effect (F(1,37) = 10.02, *P* < .01), showing a reduction of heart rate after the procedure. No statistically significant effects were found for the main factor GROUP (F(1,37) = .10, *P* = .76) or the interaction GROUP × TIME (F(1,37) = .30, *P* = .59). Although pairwise comparisons did not show significant differences between the control and the intervention group neither before intervention or after intervention (*P* > .63 in all cases), the control group showed a statistically significant decrease of the systolic blood pressure after the intervention (*P* < .05), whereas the intervention group showed only a nonsignificant tendency to decrease (*P* = .075).

With regard to diastolic blood pressure, the findings achieved a significant main effect GROUP (F(1,37) = 5.30, *P* < .05), revealing lower diastolic blood pressure in the intervention group than in the control group. No statistically significant effects were found for the main factor TIME (F(1,37) = .09, *P* = .76) or the interaction GROUP × TIME (F(1,37) = .28, *P* = .60). Although pairwise comparisons did not show significant changes of the diastolic blood pressure after the intervention in any of the study groups (*P* > .56 in all cases), the control group showed higher diastolic blood pressure levels than the intervention group in the preintervention assessment (*P* < .05), whereas only a nonsignificant tendency was found for the postintervention comparison (*P* = .065).

There were significant main effects GROUP (F(1,37) = 7.81, *P* < .01) and TIME (F(1,37) = 7.09, *P* < .05) for heart rate. A lower heart rate was observed in the intervention group compared to the control group and the score changes also showed lower heart rate values in the postintervention evaluation compared to baseline scores. Although the interaction GROUP × TIME did not show any statistical significance (F(1,37) = .58, *P* = .45), pairwise comparisons showed that only the control group decreased the heart rate after the intervention (*P* < .05). On the contrary, no changes were found in the intervention group (*P* = .19). Nevertheless, the control group showed higher heart rate than the intervention group both before and after the intervention (*P* < .05 in all cases).

## 4. Discussion

The present study aimed to evaluate the effects of the CV-4 technique in the regulation of the autonomic nervous system (ANS). More specifically, the objective of the study was to evaluate changes in plasma catecholamine levels, blood pressure, and heart rate in healthy adults that were randomly assigned to either an intervention group, who received the CV-4, or a control group, who underwent a placebo intervention. The present findings confirmed a reduction of norepinephrine, systolic blood pressure, and heart rate after the intervention, in both study groups. Likewise, only subjects of the control group showed an increase of dopamine blood levels after intervention.

Our results are in contrast with other studies reporting an influence of the CV-4 technique in autonomic-related parameters, such as heart rate, blood pressure, blood flow velocity, the electroencephalography alpha power, and the muscle sympathetic nerve activity [19–22]. The previously mentioned previous studies have used several tools such as microneurographic recording, skin conductance, skin temperature, or heart rate variability assessments to test the effects of the CV-4 technique in the ANS. However, to the best of authors' knowledge, this is the first study to assess the immediate effects of CV-4 in the plasmatic catecholamine levels. The present study has addressed this issue in a different way and, therefore, the observed findings may be different. Previous research has reported that heart rate variability can be unstable in some situations and must be cautiously interpreted to characterize sympathovagal interaction [26, 27]. Other studies have not found relationships between heart rate variability and plasmatic norepinephrine levels [28]. In this sense, our design could have included catecholamines' analysis to better indicate ANS activity. It could also be argued that the lying-down position and the relaxation provided by the procedure in both groups could have had more influence in the autonomic regulation than the CV-4 technique per se. This would be in accordance with studies reporting that increments in the head-up tilt angle have been shown to increase plasma epinephrine and heart rate [29]. Plasma catecholamine activity has also been concluded to be influenced by stressors [30], and we must also take into account the influence of the procedural setting on the sample's ANS. On the other hand, previous research has also suggested that the duration of manual intervention may have a significant impact on the autonomic response [31]. According to this, higher differences between the groups may have emerged if longer procedure times had been used. This is an important area for further research, especially when no clinical present evidence has established a proper duration for the CV-4 technique. The high standard deviations found in the catecholamine levels in the present study subjects may also explain why more significant differences have not been found. Finally, our study was performed with a healthy population; the application of CV-4 in individuals with chronic pain or autonomic dysfunction or in an experimental condition using pain stimuli could have displayed different results.

In contrast with previous research, in our study, the application of the CV-4 technique was not directly related to variation in heart rate or blood pressure [21]. It has been stated that the heart rate does not quantitatively reflect the degree of autonomic activation and Ng et al. [32] have reported changes in sympathetic nerve activity without changes in heart rate or blood pressure. Mannelli et al. [33] have related changes in catecholamine plasmatic levels to the modification of the low-frequency/high-frequency ratio, which would disregard variations in changes in heart rate. In our study, both groups experienced a reduction of norepinephrine levels after intervention. Although the present findings may indicate a tendency to a higher reduction of diastolic blood pressure and heart rate in the intervention group, the absence of significant interactions does not allow us to maintain the fact that these effects are related to the technique.

Our study has some limitations that must be taken into account for the adequate interpretation of the results. Firstly, previous research has reported significant changes in the ANS after manual techniques and placebo conditions when compared to a control group with no intervention [31]. In our study the absence of a control group with no intervention does not allow us to conclude if the mere hand contact in a lying position is sufficient to elicit a different autonomic response than in a control condition. Hence, we must question whether the proposed placebo intervention is not really a powerful intervention itself and it should not be considered as a sham intervention. Secondly, catecholamine plasmatic concentration can be influenced by factors such as age, sex, nutritional condition, emotional factors, or heat exposure [34, 35]. Although some factors have been controlled in the present research, trying to homogenize the groups and procedure conditions, some other parameters, such as the nutritional or emotional conditions, have not been controlled. The statistical analyses to observe the influence of sex in the outcome measures displayed only main statistical effects not related to the technique application. Scientific literature concludes contradictory results on the topic, going from studies reporting gender variations in catecholamine levels during rest, exercise, or stressors [35, 36] to studies not reporting gender differences [37, 38]. Our results seem to be in accordance with the latter authors, although further studies with specific designs and bigger samples are needed to control all the possible influencing variables. Moreover, heart rate measurement would have been more accurate with the use of ECG.

## 5. Conclusions

The CV-4 technique does not seem to have an immediate influence on catecholamine plasmatic levels. This finding is relevant as manual techniques may have concurrent effects on pain and on the ANS activity. In this sense, manual techniques likely to regulate sympathetic activity may be of paramount importance for providing analgesia and reducing pain sensitivity. Further research on the topic is warranted to better understand the physiological mechanisms underlying the CV-4 technique and its potential effects in the autonomic and nociceptive systems.

## Figures and Tables

**Table 1:** Mean and SD of catecholamine plasmatic levels, blood pressure, and heart rate for both groups (intervention and control) in the preintervention and postintervention.

	Intervention group		Control group	
	Preintervention	Postintervention	Preintervention	Postintervention
Dopamine	53.73 (20.15)	53.63 (15.62)	36.85 (14.47)	52.35 (19.13)
Norepinephrine	119.68 (39.55)	114.00 (44.17)	113.85 (39.64)	87.65 (37.63)
Epinephrine	43.58 (20.16)	49.26 (16.27)	53.55 (14.94)	52.15 (22.13)
Systolic blood pressure	119.74 (9.50)	116.53 (8.08)	121.55 (13.12)	117.00 (15.52)
Diastolic blood pressure	74.00 (9.18)	74.26 (7.12)	81.48 (10.25)	80.50 (12.48)
Heart rate	72.39 (6.95)	70.40 (6.93)	80.95 (13.39)	77.35 (7.99)
